# Effects of Maca (*Lepidium meyenii* Walp.) on Physical Performance in Animals and Humans: A Systematic Review and Meta-Analysis

**DOI:** 10.3390/nu17010107

**Published:** 2024-12-30

**Authors:** Álvaro Huerta Ojeda, Javiera Rodríguez Rojas, Jorge Cancino-López, Guillermo Barahona-Fuentes, Leonardo Pavez, María-Mercedes Yeomans-Cabrera, Carlos Jorquera-Aguilera

**Affiliations:** 1 Núcleo de Investigación en Salud, Actividad Física y Deporte ISAFYD, Universidad de Las Américas, Viña del Mar 2531098, Chile; ahuerta@udla.cl (Á.H.O.); g.barahonafuentes@uandresbello.edu (G.B.-F.); 2 Facultad de Ciencias, Escuela de Nutrición y Dietética, Magíster en Nutrición Para la Actividad Física y el Deporte, Universidad Mayor, Santiago 8580745, Chile; javiera.rodriguezr@mayor.cl; 3 Exercise Physiology and Metabolism Laboratory, Escuela de Kinesiología, Universidad Finis Terrae, Santiago 7501015, Chile; 4 Faculty of Education and Social Sciences, Universidad Andres Bello, Viña del Mar 2520000, Chile; 5 Núcleo de Investigación en Ciencias Biológicas (NICB), Facultad de Medicina Veterinaria y Agronomía, Universidad de Las Américas, Santiago 7500975, Chile; lpavez@udla.cl; 6 Facultad de Salud y Ciencias Sociales, Universidad de Las Américas, Viña del Mar 2531098, Chile; maria.yeomans@edu.udla.cl; 7 Facultad de Ciencias, Escuela de Nutrición y Dietética, Universidad Mayor, Santiago 8580745, Chile; carlos.jorquera@mayor.cl

**Keywords:** *Brassicaceae*, in vitro techniques, in vivo techniques, physical performance

## Abstract

Background: *Lepidium meyenii* Walp. (LmW), known as maca, has been shown to increase physical performance. However, the effect size (ES) of LmW on the different manifestations of physical performance has not yet been described. Objectives: To examine and qualitatively describe the studies published up to 2024 that employed LmW supplementation to increase physical performance in animal and human experimentation. In addition, the ES associated with the different interventions was calculated. Methods: The research followed PRISMA^®^ guidelines for systematic reviews and meta-analyses, using Web of Science, Scopus, SPORTDiscus, PubMed, and MEDLINE databases until 2024. Randomized controlled studies with a pre- and post-test design, conducted both in vitro and in vivo in animals and humans, were included. Methodological quality assessment was performed using the CAMARADES tool for animal studies and the Newcastle Ottawa Scale for human studies. The main variables were the forced swimming test (FST), the rota-rod test (RRT), the grip strength test (GST), blood lactic acid (BLA), and lactic acid (LA). The analysis was conducted with a pooled standardized mean difference (SMD) through Hedges’ g test (95% CI). Results: Twenty-one studies were included in the systematic review and sixteen in the meta-analysis. They revealed a large effect for all outcomes (SMD: FST = 2.26, RRT = 6.26, GST = 5.23, LA = −1.01, and BLA = −1.70). Conclusions: The phytochemical compounds of LmW, mainly macamides, increase physical performance, showing a greater effect at higher doses (dose–response effect).

## 1. Introduction

Over time, science has studied the effects of plants and fruits on physical performance, both naturally occurring and synthesized [1,2,3]. For example, it has been shown that Ginseng (*Panax ginseng*), a root used in traditional Asian medicine, improves physical performance, especially in tests using over 70% of maximum oxygen consumption (VO_2max_) until fatigue, either with acute or chronic ingestion effects [1]. Also, the Rhodiola plant (*Rhodiola rosea*), traditional in Nordic, Eastern European, and Asian medicine, has been used for its adaptogenic properties, showing increased physical performance and work productivity and benefits for gastrointestinal diseases and depression, among others [2]. Specifically, Rhodiola supplementation has shown beneficial effects in delaying time to fatigue in maximal incremental efforts and also in cyclo-ergometer time trails [4]. Beet (*Beta vulgaris*) has also been recognized for its effects on physical performance [5]. In fact, Domínguez et al. [5] showed that both acute and chronic supplementation improved ventilatory parameters such as VO_2max_ and anaerobic thresholds (VT2) [5].

Maca is another natural supplement that has gained popularity as an ergogenic nutritional aid worldwide [6]. Specifically, *Lepidium meyenii* Walp. (LmW) is a plant native to the Peruvian Andes, cultivated for more than 2000 years at extreme altitudes, between 3500 and 4500 m above sea level, where other plant species do not survive [7]. A member of the *Brassicaceae* family, LmW is characterized by its hypocotyl root, which can vary in color (yellow, red, and black). This root, used fresh or in powdered form and traditionally consumed in the form of juices, soups, and extracts, is rich in fiber, proteins, essential amino acids, fatty acids, and minerals such as iron, calcium, and zinc, making it a valuable food [7]. Its bioactive compounds, such as glucosinolates, macamides, and macaenes, give it antioxidant [8], anti-inflammatory, and neuroprotective properties [6,9]. These metabolites have also been shown to improve fertility and sexual desire, positioning LmW as a plant with high therapeutic potential in the field of reproductive and general health [7,9].

Similarly, in vitro and in vivo animal research has shown that LmW supplementation reduces fatigue, increases physical performance, and improves antioxidant capacity [8] as indicated by biochemical parameters associated with physical exercise [10,11]. This has led athletes to search for natural ergogenic aids to explore the effects of supplements such as LmW to improve physical performance, post-exercise recovery, and injury prevention [12,13]. Specifically, some human research has shown positive effects of LmW supplementation on specific physical tests such as cardiovascular endurance [14]. In addition, previous studies have shown that a higher dose of LmW has a greater effect on cellular oxidative stress [8]. However, despite the existing evidence, the effect size (ES) of different LmW strains and doses on physical performance in animals and humans is unknown.

Consequently, the primary objective of this systematic review and meta-analysis was to review studies published up to 2024 that have investigated the use of LmW as a nutritional ergogenic aid in animals and humans. A secondary objective was to determine the ESs of the different doses administered to identify patterns and guide future research.

## 2. Materials and Methods

This systematic review and meta-analysis were conducted under established guidelines for systematic reviews and meta-analyses [15]. To assess the risk of bias in the studies, the Collaborative Approach for the Analysis of Animal Data from Experimental Studies (CAMARADES) was used [16]. Also, the Newcastle Ottawa Scale was used to assess the methodological quality of human research (NOS) [17]. The protocol for this review is registered in PROSPERO (CRD42024586234).

### 2.1. Selection Criteria

The search for studies was carried out following the principles stipulated for systematic reviews and meta-analyses [15], using the PICOS model, which defined (i) the population (in vitro and in vivo, both in humans and animals), (ii) the intervention (LmW supplementation and its impact on physical performance), (iii) the comparators (a control group without supplementation and an experimental group with LmW supplementation, with pre- and post-intervention assessments), (iv) the positive or negative outcomes associated with physical performance variables, and (v) the quasi-experimental study design with control and experimental groups. Studies that did not meet these criteria were excluded from the review and meta-analysis. In case of any discrepancy between the authors’ evaluations at the time of the search, the discrepancy was resolved through discussion until a consensus was reached.

### 2.2. Search Strategy and Information Sources

An exhaustive search was conducted in the electronic databases Web of Science (WoS), Scopus, MEDLINE, PubMed, and SPORTDiscus. The search included studies published from inception through October 2024. The search used key terms such as [(“*Lepidium meyenii* walp.” OR “Maca” OR “Macamides” OR “Lepidium peruvianum” OR “Ginseng andean” OR “Ginseng Peruvian” OR “Ayak Chichira” OR “Ayak Willku” OR “Black maca” OR “Red maca” OR “Yellow maca” OR “Maca polysaccharide” OR “Maca powder” OR “Maca extract” OR “Glucosinolates of maca” OR “Peruvian maca”) AND (”sport performance” OR “physical performance”)], combined with Boolean operators (AND/OR). The RefWorks bibliographic manager was used to manage the search results. Two authors performed the initial search (A.H.O, and J.R.R.), and three evaluated the studies for inclusion (J.R.R., A.H.O., and G.B-F.).

### 2.3. Data Extraction

Data extracted from the selected studies included author, year, publication type, sample size, type of intervention, dependent and independent variables (focusing on LmW supplementation and physical performance), outcomes, and characteristics of the control and experimental groups. Data extraction was performed independently by the review authors, and any missing data were requested from the corresponding authors of each article. When there was no response, values were estimated from publications’ graphs. Discrepancies between authors were resolved by discussion. Data were organized in Excel spreadsheets and analyzed using Review Manager software (version 5.4, Cochrane Collaboration, Copenhagen, Denmark). The classification of studies in the systematic review and meta-analysis included the following ranges: low dose: 100–300 mg/kg, medium dose: 400–800 mg/kg, and high dose > 900 mg/kg [8,18].

### 2.4. Risk of Publication Bias Across Studies

Publication bias is a critical problem generated when published studies’ results do not adequately represent all the results obtained. This phenomenon can bias the understanding of a subject and have serious consequences on the validity of the conclusions and, sometimes, on decision-making. Egger’s test was used for the studies included in the meta-analysis to demonstrate the risk of publication bias (*p* ≤ 0.05) [19]. Graphs were generated to visualize the overall effect, supplemented by Egger’s test to confirm or rule out publication bias.

### 2.5. Assessment of Methodological Quality and Risk of Bias in Individual Studies

For animal studies, methodological quality and risk of bias were assessed using the CAMARADES checklist [16], based on ten criteria: peer review, temperature control, randomization, allocation concealment, blinded outcome assessment, no use of anesthetics with intrinsic properties, adequate animal models, sample size calculation, compliance with animal welfare regulations, and absence of conflicts of interest. The studies were classified as low quality (1–4 points), moderate (5–7 points), and high quality (8–10 points). For human studies, methodological quality and risk of bias were assessed using the NOS [17], explicitly designed for cross-sectional research, to evaluate the quality of the research [20]. The NOS assesses quality by considering content, design, and interpretation and has demonstrated high levels of reliability and validity compared to alternative scales [21]. The scale consists of three dimensions: selection, comparability, and outcome. The sample’s representativeness is assessed through seven categories: justification of sample size, comparability between respondents and non-respondents, determination of exposure, comparability based on study design or analysis, assessment of outcome, and adequacy of statistical analysis. If the study meets specific conditions, it can receive nine stars. The screening dimensions can receive a maximum of four stars, the comparability dimensions a maximum of two stars, and the results a maximum of three stars [20].

### 2.6. Statistical Analysis and Results Synthesis

The meta-analysis included studies that met the established criteria, analyzing the effects of LmW supplementation on physical performance through comparisons between the experimental and control groups. The variables considered were physical performance measures such as the forced swimming test (FST), the grip strength test (GST), the rota-rod test (RRT), and blood lactic acid (BLA), and other markers of physical performance. The analysis was performed with Review Manager software (version 5.4). The standardized mean difference (SMD) was used to assess the effect size (ES) of the intervention [22], interpreted according to Cohen’s criteria: trivial (<0.2), small (0.2–0.5), moderate (0.5–0.8), and large (>0.8) [23]. To assess between-study heterogeneity, the I^2^ statistic was calculated, indicating the total observed variation due to factors beyond chance, with low (25–50%), moderate (50–75%), and high (>75%) levels of inconsistency [24].

## 3. Results

### 3.1. Study Selection

**Figure 1:**
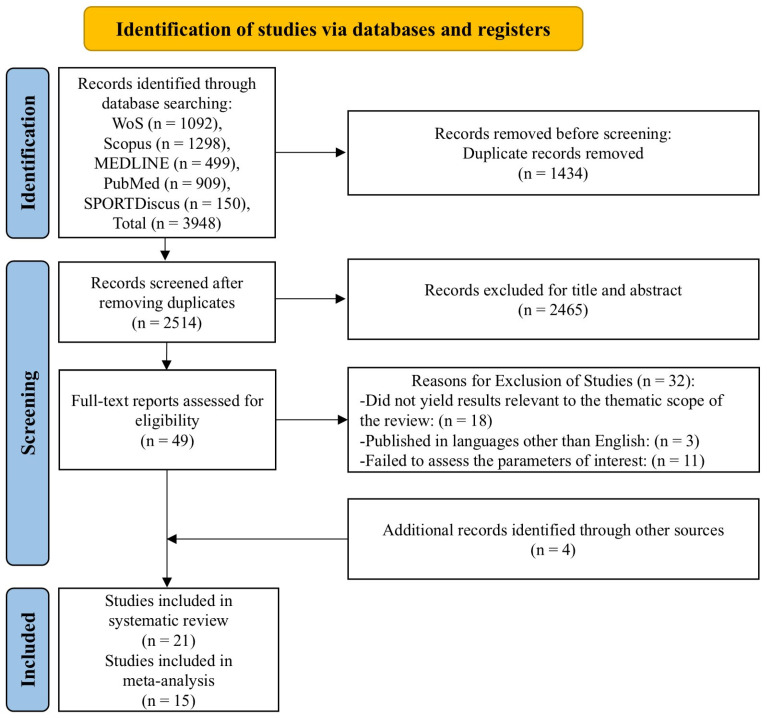
Search strategy and study selection. PRISMA flow diagram of articles that were selected.

The database search identified 3948 articles, of which 1434 were duplicates. After a review of the titles and abstracts, 49 studies were selected for in-depth analysis. Of these 49 studies, 32 were excluded because they did not meet the inclusion criteria. Additionally, four studies were incorporated. The systematic review included 21 articles, of which 16 were subjected to meta-analysis. The search strategy and study selection process are presented in Figure 1 [25].

Of the 21 studies, 17 were conducted on animals [10,11,18,26,27,28,29,30,31,32,33,34,35,36,37,38,39], while 4 involved humans [14,40,41,42]. The characteristics of the studies, the doses of LmW administered, and the results related to post-meal physical performance are detailed in Table 1.

### 3.2. Assessment of Methodological Quality of Individual Studies

Concerning animal studies, when assessing the methodological quality of the 17 studies selected for the systematic review, only the study by Orhan et al. [33] presented a “high” methodological quality. The remaining 16 studies had a “medium” methodological quality [10,11,18,26,27,28,29,30,31,32,34,35,36,37,38,39].

Concerning the human studies, when assessing the methodological quality of the four studies selected for the systematic review, two studies had a “high” methodological quality [40,41] and two an “average” methodological quality [14,42] (Table 2).

### 3.3. Meta-Analysis

Of the 21 studies selected, 15 included randomized controlled trials, test and post-test, EG and CG, and meta-analyzable outcomes [10,11,18,27,28,29,30,31,32,34,35,36,37,38,39]. These 15 studies were meta-analyzed in five physical performance outcomes: the forced swimming test (FST) [10,11,18,27,28,29,30,31,32,34,35,36,37], the rota-rod test (RRT) [38,39], the grip strength test (GST) [38,39], lactic acid (LA) [11,34,35] and blood lactic acid (BLA) [18,27,28,29,31,37,39].

### 3.4. Publication Bias

**Figure 2:**
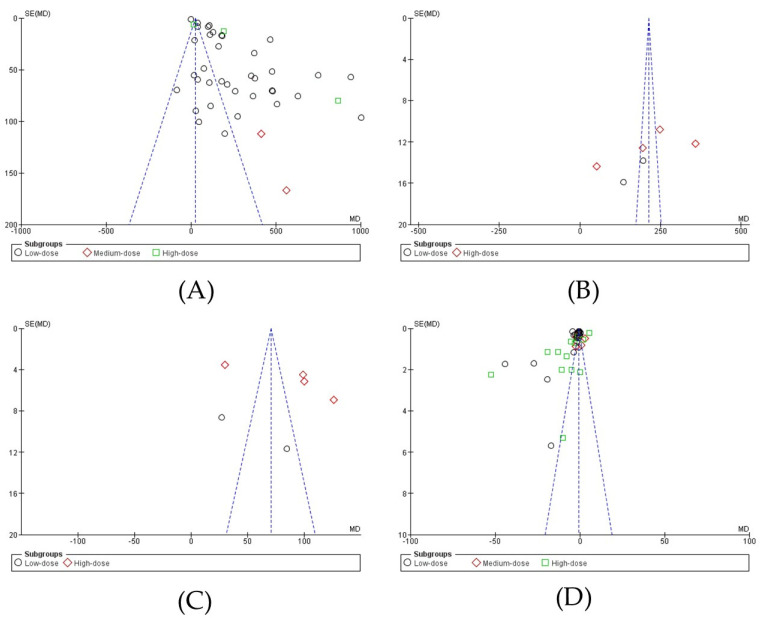
The standard error for the forced swimming test (panel (**A**)), rota-rod test (panel (**B**)), grip strength test (panel (**C**)), and blood lactic acid (panel (**D**)). SE: standard error; SMD: standardized median difference.

The presence of publication bias in the 15 meta-analyzed studies was assessed using Egger’s statistical test (*p* < 0.05) [19]. Funnel plots were created to interpret the overall effect, and Egger’s statistical test was applied to confirm or refute publication bias. Egger’s analysis revealed publication bias in the four meta-analyzed outcomes: (A) FST: z = 11.67, *p* < 0.00001 [10,11,18,27,28,29,30,31,32,34,35,36,37,38,39]; (B) RRT: z = 4.44, *p* < 0.00001 [38,39]; (C) GST: z = 4.47, *p* < 0.00001 [38,39]; and (D) BLA: z = 6.48, *p* < 0.00001 [11,18,27,28,29,31,34,35,36,37,39] (Figure 2A–D).

### 3.5. Effect of LmW on Forced Swimming Test

**Figure 3:**
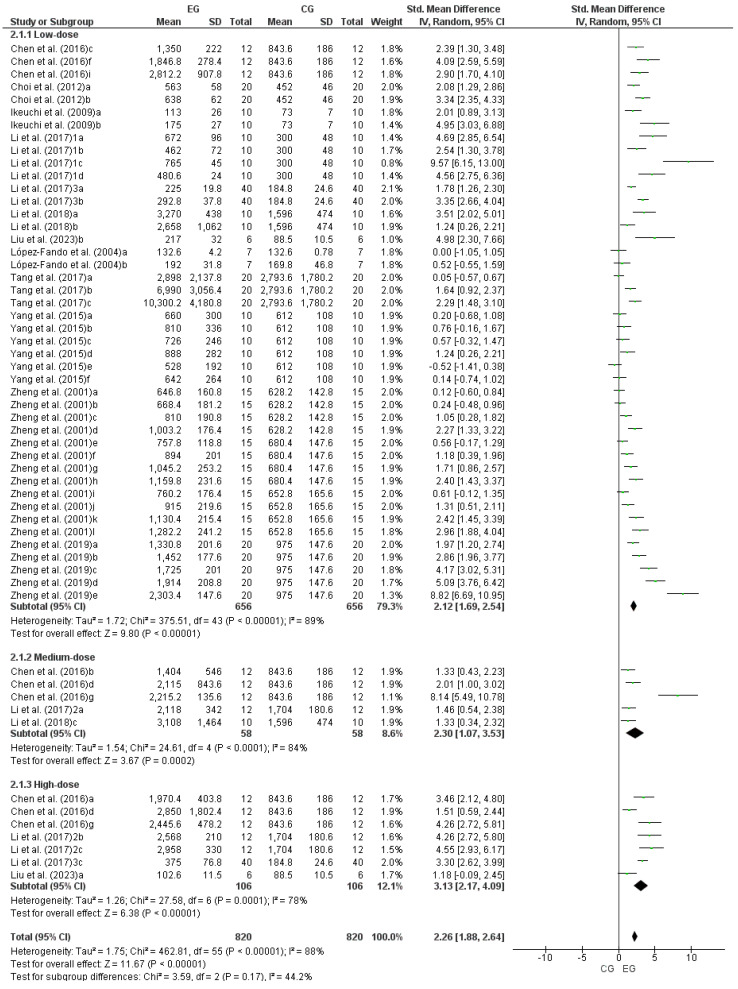
Forest plot comparing the effects of LmW on forced swimming test [10,11,18,27,28,29,30,31,32,34,35,36,37].

Thirteen studies were considered for this analysis [10,11,18,27,28,29,30,31,32,34,35,36,37]. Specifically, since the study by Chen et al. [18] considered nine EGs, it was considered as nine independent studies (a, b, c, d, e, f, g, h, and i). For the same reason, the study of Choi et al. [10] was considered as two independent studies (a and b) and, Ikeuchi et al. [11] was treated as two independent studies (a and b). Li et al. [27] as four independent studies (a, b, c, and d); Li et al. [28], Li et al. [29], and Li et al. [30] as three independent studies (a, b and c, respectively); Liu et al. [31] and López-Fando et al. [32] as two independent studies (a and b, respectively); Tang et al. [34] and Yang et al. [35] as three independent studies (a, b, and c, respectively); Zheng et al. [36] as twelve independent studies (a, b, c, d, e, f, g, h, i, j, k, and l); and Zheng et al. [37] as five independent studies (a, b, c, d, and e). Consequently, to calculate the effect of LmW on FST, this meta-analysis considered 56 comparisons as independent studies. Of these, 44 comparisons corresponded to low doses of LmW, five to medium doses, and seven to high doses of LmW. Figure 3 shows a large effect of low, medium, and high doses of LmW on physical performance in the FST (low dose: DME = 2.12; IC = 95%: 1.69–2.54; *p* < 0.00001; medium dose: DME = 2.30; IC = 95%: 1.07–3.53; *p* < 0.00001; high dose: DME = 3.13; IC = 95%: 2.17–4.09; *p* < 0.00001; total: DME = 2.26; IC = 95%: 1.88–2.64; *p* < 0.00001). The meta-analysis showed a high heterogeneity among the reviewed studies, both for comparisons between doses and in the total analysis (low dose: I^2^ = 89%; *p* < 0.00001; medium dose: I^2^ = 84%; *p* < 0,00001; high dose: I^2^ = 78%; *p* < 0.00001; total: I^2^ = 88%; *p* < 0.00001).

### 3.6. Effect of LmW on the Rota-Rod Test

**Figure 4:**
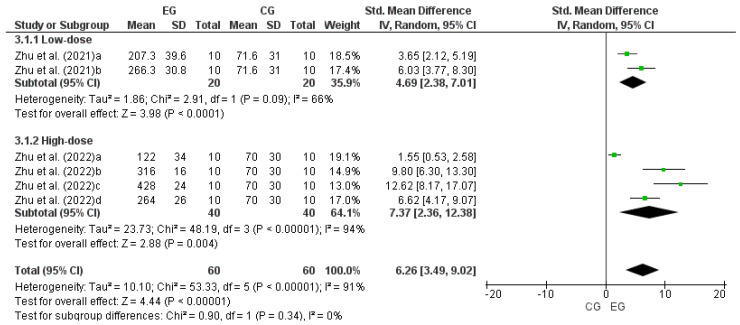
Forest plot comparing the effects of LmW on the rota-rod test [38,39].

Two studies were considered for this analysis [38,39]. Specifically, since the study had two EGs, Zhu et al. [38] was considered as two independent studies (a and b). For the same reason, Zhu et al. [39] was considered as four independent studies (a, b, c, and d). Consequently, to calculate the effect of LmW on RRT, this meta-analysis considered six comparisons as independent studies. Of these, two comparisons correspond to low and 4 to high doses of LmW. Figure 4 shows a large effect of low and high doses of LmW on physical performance in RRT (low dose: DME = 4.69; IC = 95%: 2.38–7.01; *p* < 0.00001; high dose: DME = 7.37; IC = 95%: 2.36–12.38; *p* < 0.00001; total: DME = 6.26; IC = 95%: 3.49–9.02; *p* < 0.00001). Meta-analysis showed moderate heterogeneity for comparison of low-dose LmW studies (I^2^ = 66%; *p* = 0.09) and a high heterogeneity for comparison of studies with high doses of LmW (I^2^ = 94%; *p* < 0.00001). The comparison of the totality of studies showed a high heterogeneity. Hence, it was impossible to compare all the studies (I^2^ = 91%; *p* < 0.00001).

### 3.7. Effect of LmW on the Grip Strength Test

**Figure 5:**
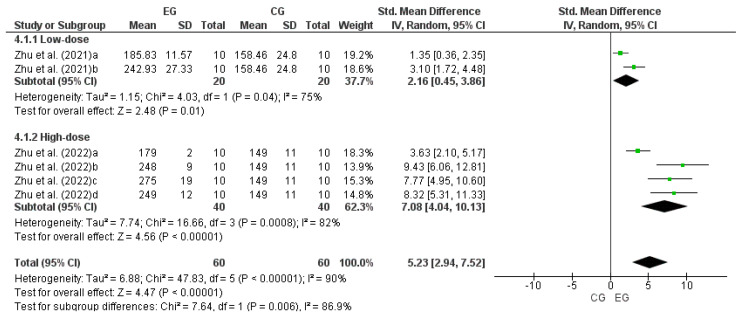
Forest plot comparing the effects of LmW on grip strength test [38,39].

Similar to the RRT analysis, the study by Zhu et al. [38] was considered as two independent studies (a and b), and the study by Zhu et al. [39] as four independent studies (a, b, c and d). Therefore, to calculate the effect of LmW on GST, six comparisons were performed as independent studies: 2 low-dose and four high-dose comparisons of LmW. Figure 5 shows a large effect of low and high doses of LmW on physical performance in GST (low dose: DME = 2.16; IC = 95%: 0.45–3.86; *p* = 0.01; high dose: DME = 7.08; IC = 95%: 4.04–10.13; *p* < 0.00001; total: DME = 5.23; IC = 95%: 2.94–7.52; *p* < 0.00001). The meta-analysis showed a high heterogeneity among the reviewed studies, both for comparisons between doses and in the total analysis (low dose: I^2^ = 75%; *p* = 0.04; high dose: I^2^ = 82%; *p* < 0.0008; total: I^2^ = 90%; *p* < 0.00001).

### 3.8. Effect of LmW on Blood Lactic Acid

**Figure 6:**
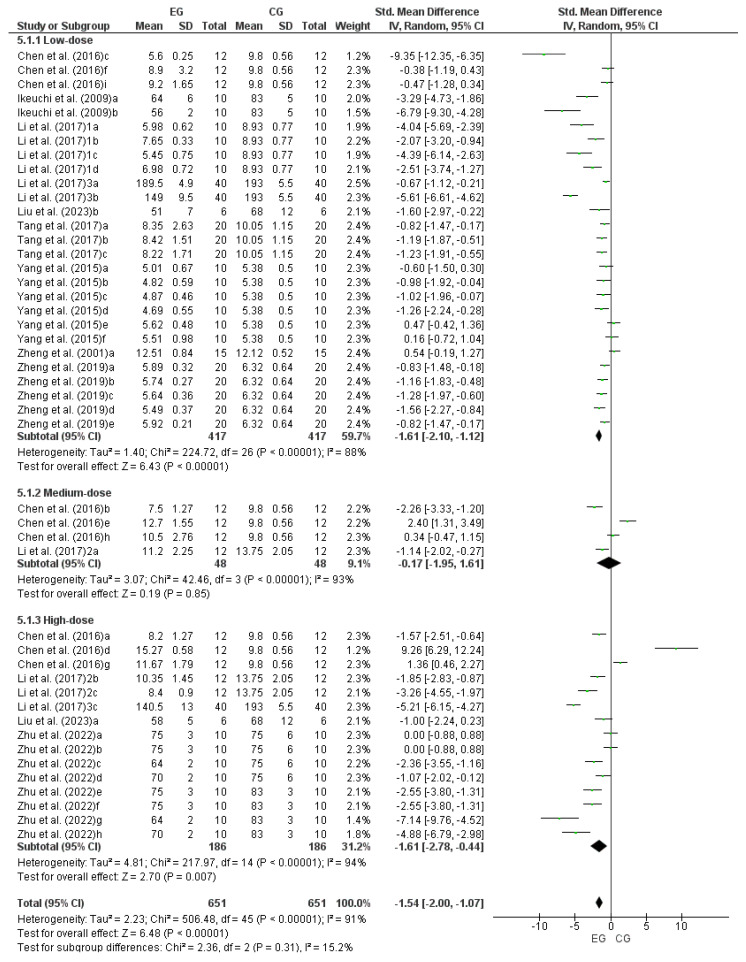
Forest plot comparing the effects of LmW on blood lactic acid [11,18,27,28,29,31,34,35,36,37,39].

Eleven studies were included in this analysis [11,18,27,28,29,31,34,35,36,37,39]. Since Chen et al. [18] considered nine EG, it was treated as nine independent studies (a, b, c, d, e, f, g, h, and i), Ikeuchi et al. [11] was treated as two independent studies (a and b), Li et al. [27] was considered as four independent studies (a, b, c and d), Li et al. [28] and Li et al. [29] as three independent studies (a, b, and c, respectively), Liu et al. [31] was considered as two independent studies (a and b), Tang et al. [34] was divided into three independent studies (a, b, and c), Yang et al. [35] into six (a, b, c, d, e, and f), Zhen et al. [36] was considered as one study (a), Zheng et al. [37] was considered as five independent studies (a, b, c, d and e); and Zhu et al. [39] was considered as eight independent studies (a, b, c, d, e, f, g and h). Consequently, to calculate the effect of LmW on BLA, this meta-analysis considered 46 comparisons as independent studies. Of these, 27 comparisons corresponded to low-dose LmW, four to medium-dose LmW, and 15 to high-dose LmW. Figure 6 shows a large effect of both low and high doses of LmW on BLA concentrations, while the medium dose showed a trivial effect on this blood marker (low dose: DME = −1.61; IC = 95%: −2.10–−1.12; *p* < 0.00001; medium dose: DME = −0.17; IC = 95%: −1.95–−1.61; *p* = 0.85; high dose: DME = −1.61; IC = 95%: −2.78–−0.44; *p* = 0.007; total: DME = −1.54; IC = 95%: −2.00–−1.07; *p* < 0.00001). The meta-analysis showed a high heterogeneity among the reviewed studies, both for comparisons between doses and in the total analysis (low dose: I^2^ = 88%; *p* < 0.00001; medium dose: I^2^ = 93%; *p* < 0.00001; high dose: I^2^ = 94%; *p* < 0.00001; total: I^2^ = 91%; *p* < 0.00001).

## 4. Discussion

### 4.1. Effect of LmW on Aerobic Test

The study results showed that supplementation with LmW, regardless of the type and format used, increases the time to exhaustion, following a dose-dependent pattern. For example, Choi et al. [10] showed that only the use of a high dose of lipid-free LmW extract (0.1 g 10 mL/kg) increased the time to fatigue (*p* < 0.05). Likewise, the results of the systematic review showed that the ingestion of LmW as NBH and benzylglucosinolate, in medium and high doses, showed significant increases in time to exhaustion in the FST (*p* < 0.05) [10,11,18,31,32,33,35,36,37]. On the other hand, in those studies that used LmW polysaccharides and water-soluble polysaccharides, the results were significantly better, independent of the dose used [18,27,28,34]. Also, Chen et al. [18] showed that the groups of mice supplemented with LmW water-soluble polysaccharides had significantly longer time to fatigue for all doses compared to mice receiving placebo. This background would imply that LmW polysaccharides would be more effective in delaying the onset of fatigue and, consequently, increasing physical performance the higher the dose administered.

The peripheral and central mechanisms that LmW possesses to reduce fatigue and improve performance in aerobic tests are not yet established, and there are no certainties. However, from a physiological point of view, fatigue is associated with the inability to sustain a given task [43]. From an energetic perspective, this inability to maintain a task is related to a reduced muscular capacity to maintain a rate of ATP resynthesis [43]. One hypothesis is that LmW supplementation positively modulates mitochondria, essential for maintaining skeletal muscle energy homeostasis [44]. Within this mitochondrial homeostasis, the peroxisome proliferator (PPARα) plays a key role in regulating the utilization of fatty acids as an energy substrate in the cell by increasing gene expression of the fatty acid oxidation enzyme (FAO) [45]. In addition, the estrogen-related receptor (ERR) activates mitochondrial energy metabolism by activating the FAO enzyme, the tricarboxylic acid cycle (TCA), the electron transport chain, and oxidative phosphorylation [46]. In this regard, the research of Wan et al. [47] observed that supplementation with black maca activated the PPARα signaling pathway, enhancing fatty acid B-oxidation, inhibiting lipogenesis, and increasing TCA flux.

Along these lines, peroxisome proliferator-activated receptor gamma proliferator-activated receptor-1 coactivator-1 (PGC-1α), which is activated by protein kinase (the latter activated by adenosine monophosphate [AMPK]), has been observed to act on multiple nuclear receptors to increase oxidative metabolism and mitochondrial biogenesis; these actions result in increased mitochondrial density, increased type I and type II fibers, and enhanced fatigue tolerance [48]. In this regard, in vitro investigations in C2C12 cells by Yi et al. [49] and Chen et al. [50] have concluded that LmW supplementation increases AMPK levels. Consequently, this increase in AMPK concentrations emerges as one of the factors that would favor increased performance in aerobic tests. However, more evidence is needed, specifically in humans.

**Figure 7:**
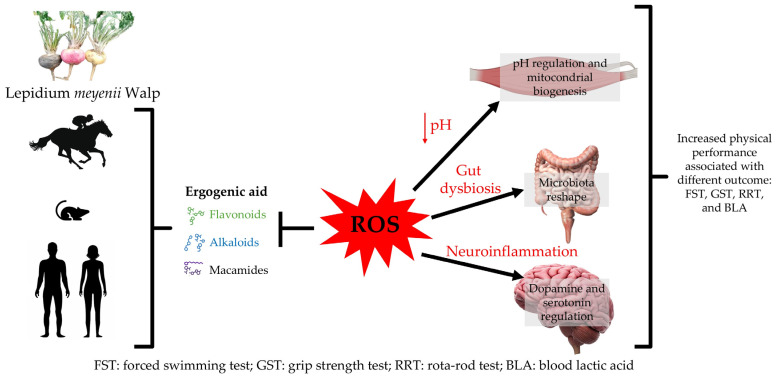
Effect of *Lepidium meyenii* Walp. (LmW) bioactive compounds on physical performance.

On the other hand, evidence has shown that an increase in skeletal muscle mitochondrial density improves the rate of fat oxidation and decreases the carbohydrate oxidation rate, allowing physical exercise to be performed for more extended periods and at high VO_2max_ percentages [51]. It is also a fact that the energy capacity of the aerobic system is greater than that of the glycolytic system so that energy production can be sustained for a prolonged period [52]. Therefore, a more efficient aerobic system reduces fatigue during exercise. Zhu et al. [38] used LmW extract on C2C12 cells, observing that the treated groups improved mitochondrial quality, mainly by blocking mitochondrial degradation when subjected to H2O2 treatment. Thus, the antioxidant capacity of LmW emerges as another factor that would allow a better performance in aerobic tests (Figure 7).

Another possible mechanism of action of LmW to improve performance in aerobic tests could be related to the modulation of the intestinal microbiota [53]. Some researchers have shown that MCP supplementation increases exercise capacity and decreases fatigue in mice, mainly by increasing the abundance and activity of Firmicutes, Actinobacteriotes, and Lactobacillus [53], the abundance and capacity of which allow metabolic regulation and better adaptation to exercise. In this context, we show Figure 7, which illustrates the possible mechanisms by extracts and preparations of LmW through its bioactive components, such as flavonoids, alkaloids, and macamides, which allow the control of cellular oxidative stress, delaying fatigue and favoring physical performance through mitochondrial biogenesis, changes in gut microbiota, and the regulation of brain dopamine and serotonin levels—in red are the suggested mechanisms by which increased ROS induce fatigue.

Finally, from a cerebral point of view, central fatigue during exercise is strongly related to increased iNOS, ROS, the 5-HIAA/5-HT metabolic pathway, and proinflammatory factors such as IL-1β and IL-6 [54,55]. Indeed, history has shown that an increase in 5-HT is associated with premature central fatigue during exercise [56]. In this sense, Zhu et al. [56], after supplementing rats with LmW in NBH format, concluded that LmW supplementation decreases iNOS, ROS, and 5-HIAA/5-HT levels and increases dopamine levels (Figure 7). Therefore, based on the above, LmW supplementation could regulate fatigue centrally and peripherally, improving performance in aerobic-dominated tests. However, these pathways need conclusive evidence in humans.

### 4.2. Effect of LmW on Glycolytic Test

#### 4.2.1. Effect on Muscle Lactic Acid Levels

Evidence indicates that LmW administration may reduce lactic acid (LA) accumulation in muscles during high-intensity exercise, possibly due to its antioxidant and adaptogenic properties [14]. Previous research has shown that bioactive compounds present in LmW, such as macamides and macaenes, mitigate exercise-induced oxidative stress by neutralizing reactive oxygen species (ROS) generated during intense muscle contraction [7]. This antioxidant effect could favor intramuscular pH recovery, limit fatigue by reducing AL accumulation, facilitate faster muscle recovery, and improve the efficiency of anaerobic metabolism [57]. Accordingly, our results suggest that LmW primarily improves high-intensity and prolonged exercise tolerance by acting as an intracellular buffer (Figure 7).

#### 4.2.2. Effect on Lactic Acid in Blood

Our results also showed reduced blood lactic acid (BLA) levels in participants supplemented with LmW. These data support the hypothesis of LmW’s positive effect on regulating systemic BLA concentrations. Furthermore, the findings are consistent with the results reported by Amir et al. [2], who found that herbal adaptogens can improve blood lactate clearance efficiency, which is crucial for maintaining sustained performance during high-intensity activities. The decrease in BLA suggests that LmW improves the body’s ability to recycle lactate through gluconeogenesis in the liver, which could extend exercise time to fatigue [58]. From a physical performance standpoint, the ability of LmW to regulate systemic BLA concentrations is relevant for athletes seeking to optimize their performance in predominantly anaerobic, high-intensity sports where lactate control is a limiting factor for physical performance [41].

### 4.3. Effect of LmW on Strength Test

Regarding using LmW as a nutritional ergogenic agent for increasing muscle strength, the results suggest that, regardless of the type and administration format, a dose-dependent response is observed, translating into an increase in grip strength capacity [38,39]. In this context, Zhu et al. [38] observed that, after 28 days of supplementation with LmW, both experimental groups (EG1 and EG2) presented a significant increase in the time (s) of resistance in the GST compared to the control group (*p* < 0.05). Similarly, Zhu et al. [39] observed that, after 28 days of LmW supplementation, there was a significant increase in the time of resistance in the GST, evidencing a dose-dependent effect in all four experimental groups (EG1, EG2, EG3, and EG4) compared to the control group (*p* < 0.05).

From a physiological perspective, muscle contraction induces oxidative stress, especially when strenuous exercise to exhaustion is performed or evaluated [59]. The mechanisms described for these intracellular events involve an increase in the concentration of reactive oxygen species (ROS) at the intracellular level, which causes a decrease in pH, generating alterations in mRNA transcription and stability and an alteration in signal transduction within the cell [60]. Finally, this series of intracellular events culminates in contractile dysfunction, which manifests as muscle weakness and fatigue in the cell [60] (Figure 7). In contrast, LmW supplementation could reduce the concentration of ROS during muscle contraction, mainly by increasing the secretion of antioxidant enzymes such as superoxide dismutase (SOD), glutathione peroxidase (GPx), reduced glutathione (GSH), and catalase (CAT) [8]. Another mechanism that could be responsible for the increase in muscle strength after LmW supplementation is the increase in muscle diameter and myotube area. These adaptations would improve the rate of differentiation and multinucleation of C2C12 musculoskeletal cells, mainly through increased phosphorylation of Akt and mTOR [49], pathways that contribute to the development and growth of muscle mass [61].

It is well known that testosterone plays a fundamental role in the production of muscle strength, mainly due to its potent anabolic effects on skeletal muscle tissue [62]. These anabolic effects occur through positive regulation of androgen receptors (AR) [61], which increases local production of insulin-like growth factor 1 (IGF-1), thus promoting muscle hypertrophy [63]. Independently, testosterone also activates Akt, the mammalian target of rapamycin (mTOR), and ribosomal protein S6 kinase 1 (S6K1) pathways [64], which increases phosphorylation of extracellular signal-regulated kinases (ERKs) [65], resulting in increased net protein accumulation and enhanced muscle hypertrophy [66]. In this context, and concerning the possible effects of LmW on testosterone, a study by Zhang et al. [67] concluded that the compound NBH, present in LmW, enhances the viability of mouse Leydig cells (TM3) under oxidative stress conditions, thus increasing testosterone levels in mice. However, it is necessary to investigate further the possible mechanisms of LmW’s action on testosterone levels and muscle strength in humans.

### 4.4. Limitations

The systematic review and meta-analysis had the following limitations: (a) the studies that evaluated the effect of LmW on physical performance in humans were scarce, which prevented a quantitative analysis and, therefore, the effect size (ES) was not calculated for this population; (b) the five variables included in the meta-analysis showed a high probability of publication bias (*p* < 0.05), so it is essential to take these indicators into account when evaluating the impact of LmW on physical performance in humans and animals; and (c) to perform a generalized analysis of the effect of macamides on physical performance, the different forms of LmW administration were grouped under the term LmW, which could induce biases in the interpretation of the results. Consequently, verifying the bioactive components of LmW declared in each research study selected for this systematic review and meta-analysis is recommended.

## 5. Conclusions

The results showed that LmW supplementation improves physical performance, as manifested in different physical tests, with an increase in efficacy as the dose increases. It was also demonstrated that the higher the dose of LmW, the greater the effect on physical performance, manifested in increased time in the FST, RRT, and GST tests, as well as lower levels of LA and BLA.

## 6. Future Lines of Research

Currently, evidence is available on the effect of LmW in humans [14,40,41,42]. However, the limited existing information does not allow for estimating the ES of LmW on physical performance in this population. Therefore, a future line of research should focus on determining the impact of LmW, in various doses and formats, on performance in aerobic, anaerobic, and muscle strength tests.

## Figures and Tables

**Table 1:** Characteristics of the studies that connect LmW with sports performance.

Authors	Participants or Sample	Independent Variable	Dependent Variable	Supplementation Protocol	Results	Effect
Research in animals						
Bilal et al. [26]	Racehorses: EG1 (n = 6) EG2 (n = 6) CG (n = 6)	EG: MPB CG: Basal diet	PO: LA	Maca root extract powder: EG1: basal diet + 50 g/day EG2: basal diet + 75 g/day CG: basal diet	LA (mmol/L): EG1 post-test = 2.27 vs. CG post-test = 1.56; *p* = 0.18 EG2 post-test = 2.05 vs. CG post-test = 1.56; *p* = 0.18	LA (mmol/L): EG1 post-test vs. CG ↔ EG2 post-test vs. CG ↔
Chen et al. [18]	Mice: EG1 (n = 12) EG2 (n = 12) EG3 (n = 12) EG4 (n = 12) EG5 (n = 12) EG6 (n = 12) EG7 (n = 12) EG8 (n = 12) EG9 (n = 12) CG (n = 12)	EG: MPB, ME and MWP CG: PL	PO: time (s) in FST, BLA	MPB: EG1 (high dose): 1.0 g/kg EG2 (medium dose): 0.5 g/kg EG3 (low dose): 0.1 g/kg ME: EG4 (high dose): 1.0 g/kg EG5 (medium dose): 0.5 g/kg EG6 (low dose): 0.1 g/kg MWP: EG7 (high dose): 1.0 g/kg EG8 (medium dose): 0.5 g/kg EG9 (low dose): 0.1 g/kg CG: distilled water	FST (s): MPB group: EG1 = 1970.4 ± 403.8 vs. CG = 843.6 ± 186; *p* < 0.01 EG2 = 1404 ± 546 vs. CG = 843.6 ± 186; *p* > 0.05 EG3 = 1350 ± 222 vs. CG = 843.6 ± 186; *p* > 0.05 ME group: EG4 = 2850 ± 1802.4 vs. CG = 843.6 ± 186; *p* > 0.05 EG5 = 2115 ± 843.6 vs. CG = 843.6 ± 186; *p* < 0.05 EG6 = 1846.8 ± 278.4 vs. CG =843.6 ± 186; *p* > 0.05 MWP group: EG7 = 2445.6 ± 478.2 vs. CG = 843.6 ± 186; *p* < 0.01 EG8 = 2215.2 ± 135.6 vs. CG = 843.6 ± 186; *p* < 0.05 EG9 = 2812.2 ± 907.8 vs. CG = 843.6 ± 186; *p* < 0.05 BLA (mmol/L): MPB groups: EG1 = 8.20 ± 1.27 vs. CG = 9.8 ± 0.56; *p* > 0.05 EG2 = 7.5 ± 1.27 vs. CG = 9.8 ± 0.56; *p* < 0.01 EG3 = 5.60 ± 0.25 vs. CG = 9.8 ± 0.56; *p* < 0.01 ME groups: EG4 = 15.27 ± 0.58 vs. CG = 9.8 ± 0.56; *p* > 0.05 EG5 = 12.70 ± 1.55 vs. CG = 9.8 ± 0.56; *p* > 0.05 EG6 = 8.90 ± 3.20 vs. CG = 9.8 ± 0.56; *p* > 0.05 MWP groups: EG7 = 11.67 ± 1.79 vs. CG = 9.8 ± 0.56; *p* > 0.05 EG8 = 10.5 ± 2.76 vs. CG = 9.8 ± 0.56; *p* > 0.05 EG9 = 9.2 ± 1.65 vs. CG = 9.8 ± 0.56; *p* > 0.05	FST (s): MP group: EG1 vs. CG ↑ EG2 vs. CG ↔ EG3 vs. CG ↔ ME group: EG4 vs. CG ↔ EG5 vs. CG ↑ EG6 vs. CG ↔ MWP group: EG7 vs. CG ↑ EG8 vs. CG ↑ EG9 vs. CG ↑ BLA (mmol/L): MP groups: EG1 vs. CG ↔ EG2 vs. CG ↓ EG3 vs. CG ↓ MAE groups: EG4 vs. CG ↔ EG5 vs. CG ↔ EG6 vs. CG ↔ MWP groups: EG7 vs. CG ↔ EG8 vs. CG ↔ EG9 vs. CG ↔
Choi et al. [10]	Mice: EG1 (n = 20) EG2 (n = 20) CG (n = 20)	EG1 and EG2: LME CG: PL	PO: time (s) in FST	Lipid soluble maca extract: EG1: 0.03 g/kg EG2: 0.1 g/kg CG: 10 mL/kg sterile water	FST (s): EG1 = 563 ± 58 vs. CG = 452 ± 46; *p* > 0.05 EG2 = 638 ± 62 vs. CG = 452 ± 46; *p* < 0.05	FST (s): EG1 vs. CG ↔ EG2 vs. CG ↑
Ikeuchi et al. [11]	Mice: EG1 (n = 10) EG2 (n = 10) CG (n = 10)	EG1 and EG2: Benzylglucosinolate CG: Distilled water	PO: time (s) in FST, LA	Benzylglucosinolate: EG1: 0.015 mg/kg EG2: 0.03 mg/kg CG: distilled water	FST (s): EG1 = 113 ± 26 vs. CG = 73 ± 7; *p* > 0.05 EG2 = 175 ± 27 vs. CG = 73 ± 7; *p* < 0.05 LA (mg/dL): EG1 = 64 ± 6 vs. CG = 83 ± 5; *p* < 0.01 EG1 = 56 ± 2 vs. CG = 83 ± 5; *p* < 0.01	FST (s): EG1 vs. CG ↔ EG2 vs. CG ↑ LA (mg/dL): EG1 vs. CG ↓ EG2 vs. CG ↑
Li et al. [27] (1)	Mice: EG1 (n = 10) EG2 (n = 10) EG3 (n = 10) EG4 (n = 10) CG (n = 10)	EG: MP CG: PL	PO: time (s) in FST, BLA	MP-1 EG1: 100 mg/kg EG2: 20 mg/kg MP-2 EG3: 100 mg/kg EG4: 20 mg/kg CG: saline solution	FST (s): EG1 = 672 ± 96 vs. CG = 300 ± 48; *p* < 0.05 EG2 = 462 ± 72 vs. CG = 300 ± 48; *p* < 0.05 EG3 = 765 ± 45 vs. CG = 300 ± 48; *p* < 0.05 EG4 = 480.6 ± 24 vs. CG = 300 ± 48; *p* < 0.05 BLA (mmol/L): EG1 = 5.98 ± 0.62 vs. CG = 8.93 ± 0.77; *p* < 0.05 EG2 = 7.65 ± 0.33 vs. CG = 8.93 ± 0.77; *p* < 0.05 EG3 = 5.45 ± 0.75 vs. CG = 8.93 ± 0.77; *p* < 0.05 EG4 = 6.98 ± 0.72 vs. CG = 8.93 ± 0.77; *p* < 0.05	FST (s): EG1 vs. CG ↑ EG2 vs. CG ↑ EG3 vs. CG ↑ EG4 vs. CG ↑ BLA (mmol/L): EG1 vs. CG ↓ EG2 vs. CG ↓ EG3 vs. CG ↓ EG4 vs. CG ↓
Li et al. [28] (2)	Mice: EG1 (n = 12) EG2 (n = 12) EG3 (n = 12) CG (n = 12)	EG: MP CG: PL	PO: time (s) in FST, BLA	MP EG1: 500 mg/kg EG2: 1000 mg/kg EG3: 2000 mg/kg CG: distilled water	FST (s): EG1 = 2118 ± 342.0 vs. CG = 1704 ± 180.6; *p* < 0.05 EG2 = 2568 ± 210.0 vs. CG = 1704 ± 180.6; *p* < 0.05 EG3 = 2958 ± 330.0 vs. CG = 1704 ± 180.6; *p* < 0.05 BLA (mmol/L): EG1 = 11.20 ± 2.25 vs. CG = 13.75 ± 2.05; *p* < 0.05 EG2 = 10.35 ± 1.45 vs. CG = 13.75 ± 2.05; *p* < 0.05 EG3 = 8.40 ± 0.90 vs. CG = 13.75 ± 2.05; *p* < 0.05	FST (s): EG1 vs. CG ↑ EG2 vs. CG ↑ EG3 vs. CG ↑ BLA (mmol/L): EG1 vs. CG ↓ EG2 vs. CG ↓ EG3 vs. CG ↓
Li et al. [29] (3)	Mice: EG1 (n = 40) EG2 (n = 40) EG3 (n = 40) CG (n = 40)	EG: Yellow maca root CG: PL	PO: time (s) in FST, BLA	Maca treatment: EG1: 40 mg/kg EG2: 400 mg/kg EG3: 1200 mg/kg CG: distilled water	FST (s): EG1 = 225 ± 19.8 vs. CG = 184.8 ± 24.6; *p* < 0.05 EG2 = 292.8 ± 37.8 vs. CG = 184.8 ± 24.6; *p* < 0.05 EG3 = 375 ± 76.8 vs. CG = 184.8 ± 24.6; *p* < 0.05 BLA (mmol/L): EG1 = 189.5 ± 4.9 vs. CG = 193 ± 5.5; *p* < 0.05 EG2 = 149.0 ± 9.5 vs. CG = 193 ± 5.5; *p* < 0.05 EG3 = 140.5 ± 13.0 vs. CG = 193 ± 5.5; *p* < 0.05	FST (s): EG1 vs. CG ↑ EG2 vs. CG ↑ EG3 vs. CG ↑ BLA (mmol/L): EG1 vs. CG ↓ EG2 vs. CG ↓ EG3 vs. CG ↓
Li et al. [30]	Mice: EG1 (n = 10) EG2 (n = 10) EG3 (n = 10) CG (n = 10)	EG: MP CG: PL	PO: time (s) in FST	MCP EG1: 150 mg/kg EG2: 300 mg/kg EG3: 600 mg/kg CG: distilled water	FST (s): EG1 = 3270 ± 438 vs. CG = 1596 ± 474; *p* < 0.01 EG2 = 2658 ± 1062 vs. CG = 1596 ± 474; *p* < 0.05 EG3 = 3108 ± 1464 vs. CG = 1596 ± 474; *p* < 0.01	FST (s): EG1 vs. CG ↑ EG2 vs. CG ↑ EG3 vs. CG ↑
Liu et al. [31]	Mice EG1 (n = 6) EG2 (n = 6) CG (n = 6)	EG: ME and NBH CG: PL	PO: time (s) in FST, BLA	ME EG1: 1000 mg/kg extract of maca NBH EG2: 10 mg/kg CG: distilled water	FST (s): EG1 = 102.5 ± 17.5 vs. CG = 88.5 ± 10.5; *p* > 0.05 EG2 = 217.0 ± 32.0 vs. CG = 88.5 ± 10.5; *p* < 0.05 BLA (mol/L) EG1 = 58 ± 5 vs. CG = 68 ± 12; *p* < 0.05 EG2 = 51 ± 7 vs. CG = 68 ± 12; *p* < 0.05	FST (s): EG1 vs. CG ↔ EG1 vs. CG ↑ BLA (mol/L) EG1 vs. CG ↓ EG2 vs. CG ↓
López-Fando et al. [32]	Mice: EG1 (n = 7) EG2 (n = 7) CG1 (n = 7) CG2 (n = 7)	EG1 and EG2: ME + stress by restraint in small flexible wire-mesh containers CG1 and CG2: PL + stress by restraint in small flexible wire-mesh containers	PO: time (s) in FST	ME EG1: 125 mg/kg EG2: 250 mg/kg CG1: 0.2 mL isotonic saline solution CG2: 0.2 mL isotonic saline solution without food and water	FST (s): EG1 = 132.6 ± 4.2 vs. CG1 = 132.6 ± 0.78; *p* > 0.05 EG1 = 132.6 ± 4.2 vs. CG2 = 169.8 ± 46.8; *p* < 0.05 EG2 = 192 ± 31.8 vs. CG1 = 132.6 ± 4.2; *p* < 0.05 EG2 = 192 ± 31.8 vs. CG2 = 169.8 ± 46.8; *p* > 0.05	FST (s): EG1 vs. CG1 ↔ EG1 vs. CG2 ↓ EG2 vs. CG1 ↑ EG2 vs. CG2 ↔
Orhan et al. [33]	Mice: EG1 (n = 7) EG2 (n = 7) CG1 (n = 7)	EG: MP and MP + FST CG: PL and PL + FST	PO: time in (s), FST	MP EG1: 40 mg/kg EG2: 40 mg/kg of MP + FST CG1: 1 mL of water	FST (min): EG1 = 16.10 ± 1.83; vs. CG1 = 0.00 ± 0.00; *p* > 0.05 EG2 = 11.15 ± 2.00; vs. CG1 = 0.00 ± 0.00; *p* < 0.01	FST (min): EG1 vs. CG1 ↔ EG2 vs. CG1 ↑
Tang et al. [34]	Mice: EG1 (n = 20) EG2 (n = 20) EG3 (n = 20) CG (n = 20)	EG: MP CG: PL	PO: time (s) in FST, LA	MP EG1: 100 mg/kg EG2: 50 mg/kg EG3: 25 mg/kg CG: distilled water	FST (s): EG1 = 10300.2 ± 4180.8 vs. CG = 2793.0 ± 1780.2; *p* < 0.01 EG2 = 6990.0 ± 3056.4 vs. CG = 2793.6 ± 1780.2; *p* < 0.01 EG3 = 2898.0 ± 2137.8 vs. CG = 2793.6 ± 1780.2; *p* < 0.05 LA (mmol/L): EG1 = 8.35 ± 2.63 vs. CG = 10.05 ± 1.15; *p* > 0.05 EG2 = 8.42 ± 1.51 vs. CG = 10.05 ± 1.15; *p* > 0.05 EG3 = 8.22 ± 1.71 vs. CG = 10.05 ± 1.15; *p* < 0.05	FST (s): EG1 vs. CG ↑ EG2 vs. CG ↑ EG3 vs. CG ↑ LA (mmol/L): EG1 vs. CG ↔ EG2 vs. CG ↔ EG3 vs. CG ↓
Yang et al. [35]	Mice: EG1 (n = 10) EG2 (n = 10) EG3 (n = 10) EG4 (n = 10) EG5 (n = 10) EG6 (n = 10) CG (n = 10)	EG: N-Benzyllinoleamide, N-benzyloleamide and N-benzylpalmitamide CG: PL	PO: time (s) in FST, LA	N- benzyllinoleamide EG1: 12 mg/kg EG2: 40 mg/kg N-benzyloleamide EG3: 12 mg/kg EG4: 40 mg/kg N-benzylpalmitamide EG5: 12 mg/kg EG6: 40 mg/kg CG: distilled water	FST (s): EG1 = 660 ± 300 vs. CG = 612 ± 108; *p* > 0.05 EG2 = 810 ± 336 vs. CG = 612 ± 108; *p* > 0.05 EG3 = 726 ± 246 vs. CG = 612 ± 108; *p* > 0.05 EG4 = 888 ± 282 vs. CG = 612 ± 108; *p* < 0.05 EG5 = 528 ± 192 vs. CG = 612 ± 108; *p* > 0.05 EG6 = 642 ± 264 vs. CG = 612 ± 108; *p* > 0.05 LA (mmol/L): EG1 = 5.01 ± 0.67 vs. CG = 5.38 ± 0.50; *p* > 0.05 EG2 = 4.82 ± 0.59 vs. CG = 5.38 ± 0.50; *p* < 0.05 EG3 = 4.87 ± 0.46 vs. CG = 5.38 ± 0.50; *p* < 0.05 EG4 = 4.69 ± 0.55 vs. CG = 5.38 ± 0.50; *p* < 0.05 EG5 = 5.62 ± 0.48 vs. CG = 5.38 ± 0.50; *p* > 0.05 EG6 = 5.51 ± 0.98 vs. CG = 5.38 ± 0.50; *p* > 0.05	FST (s): EG1 vs. CG ↔ EG2 vs. CG ↔ EG3 vs. CG ↔ EG4 vs. CG ↑ EG5 vs. CG ↔ EG6 vs. CG ↔ LA (mmol/L): EG1 vs. CG ↔ EG2 vs. CG ↓ EG3 vs. CG ↓ EG4 vs. CG ↓ EG5 vs. CG ↔ EG6 vs. CG ↔
Zheng et al. [36]	Mice: EG1 (n = 15) EG2 (n = 15 EG3 (n = 15) EG4 (n = 15) EG5 (n = 15) EG6 (n = 15) EG7 (n = 15) EG8 (n = 15) EG9 (n = 15) EG10 (n = 15) EG11 (n = 15) EG12 (n = 15) CG1 (n = 15) CG2 (n = 15) CG3 (n = 15)	EG: MacaForceTM AQ-1 CG: PL	PO: time in (s), FST	MacaForceTM AQ-1 7 days EG1: 4 mg/kg EG2: 10 mg/kg EG3: 20 mg/kg EG4: 40 mg/kg CG1: 10% ethanol/water solution 14 days EG5: 4 mg/kg EG6: 10 mg/kg EG7: 20 mg/kg EG8: 40 mg/kg CG2: 10% ethanol/water solution 21 days EG9: 4 mg/kg EG10: 10 mg/kg EG11: 20 mg/kg EG12: 40 mg/kg CG3: 10% ethanol/water solution	7 days FST (s) EG1 = 646.8 ± 160.8 vs. CG1 = 628.2 ± 142.8; *p* > 0.05 EG2 = 668.4 ± 181.2 vs. CG1 = 628.2 ± 142.8; *p* > 0.05 EG3 = 810 ± 190.8 vs. CG1 = 628.2 ± 142.8; *p* < 0.05 EG4 = 1003.2 ± 176.4 vs. CG1 = 628.2 ± 142.8; *p* < 0.01 14 days FST (s) EG5 = 757.8 ± 118.8 vs. CG2 = 680.4 ± 147.6; *p* > 0.05 EG6 = 894 ± 201 vs. CG2 = 680.4 ± 147.6; *p* < 0.05 EG7 = 1045.2 ± 253.2 vs. CG2 = 680.4 ± 147.6; *p* < 0.05 EG8 = 1159.8 ± 231.6 vs. CG2 = 680.4 ± 147.6; *p* < 0.05 21 days FST (s) EG9 = 760.2 ± 176.4 vs. CG3 = 652.8 ± 165.6; *p* > 0.05 EG10 = 915 ± 219.6 vs. CG3 = 652.8 ± 165.6; *p* < 0.01 EG11 = 1130.4 ± 215.4 vs. CG3 = 652.8 ± 165.6; *p* < 0.01 EG12 = 1282.2 ± 241.2 vs. CG3 = 652.8 ± 165.6; *p* < 0.05	7 days FST (s) EG1 vs. CG1 ↔ EG2 vs. CG1 ↔ EG3 vs. CG1 ↑ EG4 vs. CG1 ↑ 14 days FST (s) EG5 vs. CG2 ↔ EG6 vs. CG2 ↑ EG7 vs. CG2 ↑ EG8 vs. CG2 ↑ 21 days FST (s) EG9 vs. CG3 ↔ EG10 vs. CG3 ↑ EG11 vs. CG3 ↑ EG12 vs. CG3 ↑
	Mice: EG (n = 15) CG (n = 15)	EG: MacaForceTM AQ-2 CG: PL	PO: time (s) in FST, LA	MacaForceTM AQ-2 EG: 40 mg/kg CG: 10% ethanol/water solution	FST (s) EG = 123.6 ± 3 vs. CG = 109.8 ± 2.4; *p* < 0.01 LA post 20 min rest (mmol/L) EG = 16.62 ± 0.67 vs. CG = 12.13 ± 0.52; *p* < 0.01 LA post 50 min rest (mmol/L) EG1 = 12.51 ± 0.84 vs. CG = 6.56 ± 0.35; *p* < 0.01	FST (s) EG vs. CG ↑ LA post 20 min rest (mmol/L) EG vs. CG ↓ LA post 50 min rest (mmol/L) EG vs. CG ↓
Zheng et al. [37]	Mice: EG1 (n = 20) EG2 (n = 20) EG3 (n = 20) EG4 (n = 20) EG5 (n = 20) CG (n = 20)	EG: CME, PME, and maca tablet CG: PL	PO: time in (s), FST, BLA	CME EG1: 30 mg/kg EG2: 120 mg/kg PME EG3: 8 mg/kg EG4: 32 mg/kg Maca tablet EG5: 165 mg/kg CG: aqueous solution	FST (s): EG1 = 1330.8 ± 201.6 vs. CG = 975 ± 147.6; *p* > 0.05 EG2 = 1452 ± 177.6 vs. CG = 975 ± 147.6; *p* > 0.05 EG3 = 1725 ± 201 vs. CG = 975 ± 147.6; *p* < 0.05 EG4 = 1914 ± 208.8 vs. CG = 975 ± 147.6; *p* < 0.05 EG5 = 2303.4 ± 203.4 vs. CG = 975 ± 147.6; *p* < 0.05 BLA (mmol/L): EG1 = 5.89 ± 0.32 vs. CG = 6.32 ± 0.64; *p* < 0.05 EG2 = 5.74 ± 0.27 vs. CG = 6.32 ± 0.64; *p* < 0.05 EG3 = 5.64 ± 0.36 vs. CG = 6.32 ± 0.64; *p* < 0.05 EG4 = 5.49 ± 0.37 vs. CG = 6.32 ± 0.64; *p* < 0.05 EG5 = 5.92 ± 0.21 vs. CG = 6.32 ± 0.64; *p* < 0.05	FST (s): EG1 vs. CG ↔ EG2 vs. CG ↔ EG3 vs. CG ↑ EG4 vs. CG ↑ EG5 vs. CG ↑ BLA (mmol/L): EG1 vs. CG ↓ EG2 vs. CG ↓ EG3 vs. CG ↓ EG5 vs. CG ↓
Zhu et al. [38]	Mice: EG1 (n = 10) EG2 (n = 10) CG1 (n = 10) CG2 (n = 10)	EG: ME and caffeine CG: PL and PL + exercise	PO: time in (s), RRT, (gf); GST	ME: EG1: 10 mL/kg EG2: 10 mg/kg caffeine CG1: 10 mL/kg sterile water CG2: 10 mL/kg sterile water + exercise	GST (gf): EG1 = 185.83 ± 11.57 vs. CG = 158.46 ± 24.80; *p* < 0.05 EG2 = 242.93 ± 27.33 vs. CG = 158.46 ± 24.80; *p* < 0.01 RRT (min): EG1 = 207.3 ± 39.6 vs. CG = 71.6 ± 31.0; *p* < 0.01 EG2 = 266.3 ± 30.8 vs. CG = 71.6 ± 31.0; *p* < 0.01	GST (gf): EG1 vs. CG ↑ EG2 vs. CG ↑ RRT (min): EG1 vs. CG ↑ EG2 vs. CG ↑
Zhu et al. [39]	Mice EG1 (n = 10) EG2 (n = 10) EG3 (n = 10) EG4 (n = 10) CG (n = 10)	EG: MCP CG: PL	PO: time (s) in RRT, grams-force (gf); GST, BLA	MCP EG1: 1.0 g/kg MCP EG2: 2.0 g/kg MCP EG3: 4.0 g/kg MCP EG4: 10 mg/kg caffeine CG1: 1.0 g/kg sterile water CG2: 1.0 g/kg sterile water + Ex	RRT (s): EG1 = 122 ± 34 vs. CG1 = 70 ± 30; *p* < 0.05 EG2 = 316 ± 16 vs. CG1 = 70 ± 30; *p* < 0.01 EG3 = 428 ± 24 vs. CG1 = 70 ± 30; *p* < 0.01 EG4 = 264 ± 26 vs. CG1 = 70 ± 30; *p* < 0.01 GST (gf): EG1 = 179 ± 2 vs. CG1 = 149 ± 11; *p* < 0.01 EG2 = 248 ± 9 vs. CG1 = 149 ± 11; *p* < 0.01 EG3 = 275 ± 19 vs. CG1 = 149 ± 11; *p* < 0.01 EG4 = 249 ± 12 vs. CG1 = 149 ± 11; *p* < 0.01 BLA (μg/L): EG1 = 75 ± 3 vs. CG1 = 70 ± 2; *p* > 0.05 EG1 = 75 ± 3 vs. CG2 = 83 ± 3; *p* > 0.05 EG2 = 75 ± 3 vs. CG1 = 70 ± 2; *p* > 0.05 EG2 = 75 ± 3 vs. CG2 = 83 ± 3; *p* < 0.01 EG3 = 64 ± 2 vs. CG1 = 70 ± 2; *p* > 0.05 EG3 = 64 ± 2 vs. CG2 = 83 ± 3; *p* < 0.01 EG4 = 75 ± 6 vs. CG1 = 70 ± 2; *p* > 0.05 EG4 = 75 ± 6 vs. CG2 = 83 ± 3; *p* > 0.05 CG1 = 70 ± 2 vs. CG2 = 83 ± 3; *p* < 0.05	RRT (s): EG1 vs. CG1 ↑ EG2 vs. CG1 ↑ EG3 vs. CG1 ↑ EG4 vs. CG1 ↑ GST (gf): EG1 vs. CG1 ↑ EG2 vs. CG1 ↑ EG3 vs. CG1 ↑ EG4 vs. CG1 ↑ BLA (μg/L): EG1 vs. CG1 ↔ EG1 vs. CG2 ↔ EG2 vs. CG1 ↔ EG2 vs. CG2 ↓ EG3 vs. CG1 ↔ EG3 vs. CG2 ↓ EG4 vs. CG1 ↔ EG4 vs. CG2 ↔ CG1 vs. CG2 ↔
Research in humans						
Honma et al. [40]	Adult women (n = 55) EG (n = 27) PL (n = 28)	EG: ME + benzylglucosinolate CG: PL	PO: VAS (cm)	ME + benzyl glucosinolate EG: 200 mg/kg CG: 200 mg/kg dextrine	VAS (cm): EG pre-test vs. post-test: 7.55 ± 0.65 vs. 5.20 ± 1.48; *p* < 0.01 CG pre-test vs. post-test: 7.52 ± 0.80 vs. 5.67 ± 1.74; p < 0.01 EG post-test vs. CG post-test: 5.20 ± 1.48 vs. 5.67 ± 1.74; *p* > 0.05	VAS (cm): EG pre-test vs. test ↑ CG pre-test vs. post-test post-test vs. CG post-test ↔
Lee et al. [14]	Male elite athletes (n = 44) SA, n = 15 RSA, n = 16 FSA, n = 13	SA, RSA, and FSA: Black maca extract	PO: muscle strength (kg), muscle endurance (number of repetitions/1min), flexibility (cm), power (cm), agility (s), cardiovascular endurance (repetition)	Black maca extract SA: 5000 mg/kg RSA: 5000 mg/kg FSA: 5000 mg/kg	Left grip strength (kg): Pre SA = 43.8 ± 4.7 vs. post SA = 45.1 ± 4.0; *p* > 0.05 Pre RSA = 41.0 ± 5.9 vs. post RSA = 41.0 ± 5.0; *p* > 0,05 Pre FSA = 34.9 ± 7.5 vs. post FSA = 39.0 ± 9.9; *p* < 0.01 Right grip strength (kg) Pre SA = 46.0 ± 5.2 vs. post SA = 47.0 ± 6.3; *p* > 0.05 Pre RSA = 46.2 ± 5.8 vs. post RSA = 46.8 ± 5.4; *p* > 0,05 Pre FSA = 38.1 ± 8.1 vs. post FSA = 42.1 ± 9.8; *p* < 0.01 Sit-ups (re*p*): Pre SA = 40.2 ± 7.0 vs. post SA = 47.1 ± 7.7; *p* < 0.05 Pre RSA = 41.5 ± 9.3 vs. post RSA = 47.7 ± 8.0; *p* < 0,05 Pre FSA = 55.8 ± 11.2 vs. post FSA = 57.9 ± 8.7; *p* < 0.01 Sit-and-reach (cm): Pre SA = 8.53 ± 8.4 vs. post SA = 10.4 ± 7.2; *p* > 0.05 Pre RSA = 6.91 ± 11.4 vs. post RSA = 9.9 ± 11.1; *p* > 0,05 Pre FSA = 22.2 ± 9.5 vs. post FSA = 25.3 ± 8.1; *p* < 0.01 Long jump (cm): Pre SA = 210.5 ± 19.2 vs. post SA = 215.0 ± 15.1; *p* > 0.05 Pre RSA = 228.9 ± 14.0 vs. post RSA = 239.5 ± 12.8; *p* < 0.05 Pre FSA = 197.8 ± 33.5 vs. post FSA = 222.1 ± 32.5; *p* < 0.01 10 m shuttle run (s): Pre SA = 10.0 ± 0.6 vs. post SA = 10.5 ± 0.6; *p* < 0.05 Pre RSA = 9.1 ± 0.7 vs. post RSA = 9.5 ± 0.4; *p* < 0.05 Pre FSA = 10.0 ± 0.9 vs. post FSA = 11.0 ± 1.2; *p* < 0.01 20 m shuttle run (s): Pre SA = 33.3 ± 3.6 vs. post SA = 37.5 ± 8.0; *p* > 0.05 Pre RSA = 62.9 ± 9.4 vs. post RSA = 64.1 ± 12.6; *p* > 0.05 Pre FSA = 74.5 ± 19.3 vs. post FSA = 70.4 ± 17.1; *p* > 0.05	Left grip strength (kg): Pre SA vs. post SA ↔ Pre RSA vs. post RSA ↔ Pre FSA vs. post FSA ↓ Right grip strength (kg) Pre SA vs. post SA ↔ Pre RSA vs. post RSA ↔ Pre FSA vs. post FSA ↓ Sit-ups (re*p*) Pre SA vs. post SA ↓ Pre RSA vs. RSA ↓ Pre FSA vs. post FSA ↓ Sit-and-reach (cm) Pre SA vs. post SASA ↔ Pre RSA vs. post RSA ↔Pre FSAA vs. post FSA ↓ Long jump (cm): Pre SA vs. post SA ↔ Pre RSA vs. post RSA ↓ Pre FSA vs. post FSA ↓ 10 m shuttle run (s) Pre SA vs. post SA ↓ Pre RSA vs. RSA ↓ Pre FSA vs. post FSA ↓ 20 m shuttle run (s): Pre SA vs. post SA ↔ Pre RSA vs. post RSA ↔ Pre RSA vs. post RSA ↔
Liu et al. [41]	Healthy men EG (n = 9) CG (n = 11)	EG: Maca extract capsule CG: PL	PO: time (min) to exhaustion	Maca extract capsule: EG: 2 capsules of 2.25 g maca extract CG: 2 capsules of cornstarch	TTE (min) EG = 63.83 ± 3.44 vs. CG = 64.04 ± 2.76; *p* > 0.05	TTE (min) EG vs. CG ↔
Stone et al. [42]	Male cyclists: EG (n = 8)	EG: ME	PO: time (min); 40 km cycling time trial after 14 days of supplementation	ME EG: 2000 mg/kg	40 km trial (min): Pre ME = 57.62 ± 3.14 vs. post ME = 56.56 ± 2.68; *p* < 0.01	40 km trial (min): Pre ME vs. post ME ↓

BLA: blood lactic acid, CG: control group, cm: centimeters, CME: crude macamide extract, EG: experimental group, FST: forced swimming test, FSA: fin swimming athletes, g: grams, gf: grams-force, GST: grip strength test, g/kg: grams per kilogram, kg: kilograms, km: kilometers, LA: lactic acid, LME: liquid-soluble maca extract, ME: maca extract, mg/dL: milligrams per deciliter, mg/kg: milligrams per kilogram, mL/kg: milliliters per kilogram, mmol/L: millimole per liter, mol/L: mol per liter, ME: maca extract, MCP: maca compound preparation, MP: maca powder, MPB: maca powder blend, MWP: maca water polysaccharides, NBH: N-benzyl-9z-12z-15z-octadecenamide, PL: placebo, PME: purified maca extract, PO: principal outcome, RRT: rota-rod test, RSA: racket sports athletes, s: seconds, SA: shooting athletes, TTE: time to exhaustion, VAS: visual analog scale, μg/L: microgram per liter, ↑: increase in the variable evaluated; ↔: no statistical change in the variable evaluated; ↓:decrease of the variable evaluated.

**Table 2:** Methodological quality of animal and human studies.

Methodological Quality of Animal Studies (CAMARADES Scale)											
Authors	(1)	(2)	(3)	(4)	(5)	(6)	(7)	(8)	(9)	(10)	TOTAL
Bilal et al. [26]	*	0	0	0	0	*	*	0	*	*	5
Chen et al. [18]	*	*	*	0	0	*	*	0	*	*	7
Choi et al. [10]	*	*	*	0	0	*	*	0	*	*	7
Ikeuchi et al. [11]	*	*	0	0	0	*	0	0	*	*	5
Li et al. [27] (1)	*	*	*	0	0	*	*	0	*	*	7
Li et al. [28] (2)	*	*	*	0	0	*	*	0	*	*	7
Li et al. [29] (3)	*	*	*	0	0	*	*	0	*	*	7
Li et al. [30]	*	*	*	0	0	*	*	0	*	*	7
Liu et al. [31]	*	*	*	0	0	*	*	0	*	*	7
López-Fando et al. [32]	*	*	*	0	0	*	*	0	*	*	7
Orhan et al. [33]	*	*	*	0	0	*	*	*	*	*	8
Tang et al. [34]	*	*	*	0	0	*	*	0	*	*	7
Yang et al. [35]	*	*	*	0	0	*	*	0	*	*	7
Zheng et al. [36]	*	0	*	0	0	*	*	0	0	*	5
Zheng et al. [37]	*	*	0	0	0	*	*	0	*	*	6
Zhu et al. [38]	*	*	*	0	0	*	*	0	*	*	7
Zhu et al. [39]	*	*	*	0	0	*	*	0	*	*	7
**Methodological quality of human studies (NOS)**											
**Authors**	**(A)**				**(B)**		**(C)**			**TOTAL**	
	**(1)**	**(2)**	**(3)**	**(4)**	**(5)**	**(6)**	**(7)**	**(8)**	**(9)**		
Honma et al. [40]	*	*	*	*	*	*	*	*	0	8	
Lee et al. [14]	*	*	*	*	*	0	*	0	0	6	
Liu et al. [41]	*	*	*	*	*	*	*	0	*	8	
Stone et al. [42]	*	*	*	*	*	0	*	0	0	6	

CAMARADES scale: studies fulfilling the criteria of (1) peer-reviewed publication; (2) control of temperature; (3) random allocation to treatment or control; (4) allocation concealment; (5) blinded assessment of outcome; (6) without use of anesthetic with intrinsic properties; (7) use of animal models (not aged, diabetic, or hypertensive); (8) sample size calculation; (9) compliance with animal welfare regulations; and (10) without conflicts of interest. Methodological quality: low 1–4; medium 5–7; high 8–10. NOS: (A) selection; (B) comparability; (C) exposure/outcome; (1) sample representativeness; (2) selection of controls; (3) definition of cases and controls; (4) source of data; (5) control of major confounding factors; (6) control of additional factors; (7) assessment of exposure; (8) non-response or follow-up criteria; (9) follow-up duration. Methodological quality: low 1–3; medium 4–6; high 7–9. *: the criterion is observed in the study; 0: the criterion is not observed in the study.
